# The effect of exercise on resilience, its mediators and moderators, in a general population during the UK COVID-19 pandemic in 2020: a cross-sectional online study

**DOI:** 10.1186/s12889-022-13070-7

**Published:** 2022-04-25

**Authors:** Molly Rose Lancaster, Patrick Callaghan

**Affiliations:** 1 Conducted research at London Southbank University, 103 Borough Road, London, SE1 0AA UK; 2 Professor of Mental Health Science and Associate Pro Vice-Chancellor Research, London Southbank University, 103 Borough Road, London, SE1 0AA UK

**Keywords:** Resilience, Exercise, Quality of life, Mental health, Sleep

## Abstract

**Background:**

Resilience is central to positive mental health and well-being especially when faced with adverse events. Factors such as exercise, location, sleep, mental health, and personality are moderators and mediators of resilience. However, the impact of these factors on resilience during severe adverse events are unknown. The present study examined how the COVID-19 pandemic affected resilience and its moderators and mediators by investigating whether there was a difference in resilience and quality of life between people with varying levels of exercise, including those who changed their exercise levels pre and during a COVID-19-related lockdown, and whether location affected the relationship between levels of exercise and resilience and quality of life.

**Methods:**

Following ethical approval, a cross-sectional online survey capturing data on self-reported key moderators and mediators of resilience before and during the COVID-19 lockdown imposed on the 23rd March 2020 in the UK was distributed via social media and completed over a three week time period during July 2020 via a self-selecting sample of the general population (*N* = 85). The key moderators and mediators of resilience the survey assessed were exercise, location, life-orientation, mental health, and sleep quality. All data were self-reported.

**Results:**

Participants’ exercise intensity level increased as resilience increased (F(2,82) = 4.22, *p* = .003: Wilks’ lambda = .82, partial n^2^ = 0.09). The relationship between exercise, and resilience and quality of life was independent of sleep and mental health status pre-lockdown (*p* = .013, *p* = .027 respectively). In the face of the COVID-19 pandemic, this relationship was dependent on mental health but not sleep quality (*p* = <.001 for resilience *p* = .010 for quality of life). There were no statistically significant differences between participants living in urban or rural locations.

**Conclusion:**

Exercise is strongly correlated to resilience and during a pandemic such as COVID-19 it becomes a mechanism in which to moderate resilience. The relationship between exercise and resilience is supported by this study. The influence that a pandemic had on mental health is mediated by its effect on quality of life.

## Background

The roots of resilience theory come from the study of adversity, otherwise known as a pathogenic focus [1] concerning how individuals achieve positive health and wellbeing outcomes. The original concept of resilience was developed as a response to large-scale external change [2], such as the United Kingdom lockdown imposed on the 23rd March 2020 in response to COVID-19. Resilience is complex in nature [3], and is characterised as the process of effectively negotiating, adapting to, or managing significant sources of stress or trauma [4–7]. Positive adaptation is the ability to maintain or regain mental health, despite experiencing adversity [6]. Resilience provides people with the strength to overcome adversity and decrease the negative effects that adversity, such as a pandemic like COVID-19 can cause [8].

Research into resilience and healthcare outcomes have shown resilience and perceived quality of life (QOL) [9] are correlated with optimism [10, 11] and lower psychological stress [12, 13]. Individuals demonstrating high resilience are less likely to report adverse mental health experiences [7, 14]. The positive impact of exercise on resilience [15–18], QOL [19] and mental health has been demonstrated in clinical and general populations [12, 20, 21].

Exercise increases resilience at the core biological level; promoting secretion of neurotransmitters and endorphins to induce a state of euphoria ([22, 23]; Highes et al., 2013). Euphoria reduces dysfunctional ideation time, acting as a distraction from stressful events ([22, 23]; Lines et al., 2018; Peluso & de Andrade, 2005).

In a study of 775 adolescents, high self-esteem correlated significantly with good mental health prognoses, with a Pearson’s product-moment correlation demonstrating a significant relationship, with resilience explaining 60% of the variance [18]. However, as the positive mental effects of exercise are enhanced by the social interactions workout sessions provide ([15, 22]; Ka et al., 2015; Peluso & de Andrade, 2005), most research to date fails to control for the effect of these interactions on the outcomes of interest.. Therefore, it cannot be concluded that exercising alone produces positive mental health effects. However, as pandemic restrictions prohibited socialising, this provided a unique research opportunity to investigate the effects of exercise without any social interactions during workout sessions.

Resilience is increased by euphoria as it increases self-esteem by representing our self-rating of self-worth (Peluso & de Andrade, 2005): the biological and psychological benefits of exercise working in unison to increase resilience. Empirical evidence commonly reports exercising at preferred intensity (one’s chosen exercise level) has increased mental health benefits compared with prescribed intensity (imposed exercise level) ([24, 25]; Carter, Bastounis, Guo, Morrell, & Carter, 2019 [26];; Turner, Carter, Sach, Guo, & Callaghan, 2017). As mental health mediates resilience, this suggests the simple act of moving has an impact on one’s resilience.

Preferred intensity maybe indirectly related to improving self-esteem due to the self-controlled nature of exercise, with observed body change results being dependent on the individual choosing the intensity, thus a goal being obtained and intrinsic motivation being stimulated ([27]; Pekrun, Hall, Goetz, & Perry, 2014). Research into preferred intensity is limited; being intervention-based six to twelve-week studies. Therefore, the effect of preferred intensity upon mental health over a randomised self-motivated population is not known. As many of the resilient and continued protective effects from exercise are correlated with the continuation of exercise throughout the lifespan [19, 21], a short intervention study cannot conclude confidently about the continued effect of preferred intensity exercise and resilience. The current study targeted this limitation as all those who exercise had done so autonomously, not knowing it would be investigated for research purposes. The comparison between those who were already exercising before lockdown and those who began once lockdown was imposed gave an insight into the long-term effects of exercise on resilience and how quickly exercise can promote resilience. This is in-line with self-determination theory [27], with exercising during lockdown being intrinsically motivated and causally related to preferred intensity, which as the cited literature has suggested, maximises the effect on resilience, thus providing clearer insights into how the exercise of the population as a whole affects resilience.

To date, studies have researched resilience and its moderators in either clinical or general populations, leaving the general population under-researched, hence the current study. Despite the reviewed literature suggesting a study amongst a self-selecting sample of the population into resilience should not be impacted by poor mental health, it indicates a need to control for mental health as a co-variant. Controlling for mental health as a co-variant would strengthen the statement that exercise increases resilience in the generic population.

The lockdown imposed on the UK on the 23rd March 2020 due to COVID-19 has provided a unique opportunity for this study to investigate areas in which previous research has been limited. The adversity and life-style changes imposed [28] allows comparisons to be made against the broad population within the UK who had to adhere to rules that go against the natural biological and psychological nature of *Homo sapiens*; pack animals who require social interaction to form a social identity which mediates resilience [15, 29–31]. Adversity has included isolation, separation, financial strain, grief, and educational deficits (BBC, 2020 [32];), research shows this negatively impacts resilience [33, 34]. These studies were not conducted in a pandemic, thus, whether these constraints affect resilience similarly in a pandemic is unknown and explored in the current study. Based on data from past recessions; such as the 2008 economic crisis, where suicide rates in Europe increased by 6.5% the prognosis for mental health and wellbeing as a result of lockdown is not predicted to be positive ([35, 36]; Radio4 (BBC), 2020 [37];). As research is scarce, we do not yet know how the COVID-19 lockdown has impacted resilience or its empirical moderators.

The aim of the current study was to examine how the COVID-19 pandemic affected resilience and its moderators by investigating if there was a difference in resilience and quality of life between people with varying levels of exercise, including those who changed their exercise levels pre and during the COVID-19 pandemic, and whether location played a role in this relationship. The authors anticipated:

1. People reporting higher exercise levels would have better resilience and QOL than those reporting low and moderate exercise levels pre-COVID-19 lockdown.
2. People who improve their exercise levels during COVID-19 lockdown would have better resilience and QOL than people whose exercise levels reduced or remained the same.
3. Mental health and sleep quality would moderate the relationship between exercise levels and resilience.
4. Exercise levels and resilience would differ between people living in rural and urban environment during COVID-19.
5. Life-orientation and resilience would differ between people living in rural and urban environments.
6. The relationship between exercise levels, resilience and QOL in people living in rural and urban environments would moderated by mental health and sleep quality during COVID-19.

## Methods

### Design

The study used a cross-sectional online survey developed on Qualtrics XM (version 26).

### Participants

Following ethics approval from the University’s Research Ethics Committee, data was collected from 126 Participants over a three-week period in June 2020. Forty-one were removed due to an incomplete data set, leaving 85 participants. The participants consisted of 31 males and 54 females with the mean age of 47.04 (SD = 18.98) who accessed the survey advertised on social media. Forty percent of participants (*n* = 34) and 60% (*n* = 51) described their location as rural and urban respectively. Before the initial lockdown period 52.9, 38.8 and 8.2% of participants were very, moderately, or not active respectively and during the initial lockdown 60, 31.8 and 8.2% of participants were active, moderately active, or not active respectively.

### Questionnaires

The survey comprised measures of the following variables:

- Demographic information: age, gender, urban or rural location.
- The Connor-Davidson Resilience Scale (CD-RISC) [5]: a 25-item self-report five-point Likert scale, ranging from 0 (not true at all) to 4 (true nearly all the time) to items such as “I am able to adapt when changes occur”, designed to assess level of resilience with higher scores indicating higher resilience. The CD-RISC has a high level of internal consistency (Cronbach’s alpha = .89) and a high test-retest reliability (52.7–52.8).
- Symptom Checklist − 5 (SCL-5) [38]: a 5-item shortened version of the Hopkins Symptom Checklist, measuring anxiety, depression and their resulting adversity. The response options were measured on a four-point Likert scale from 1 (not at all) to 4 (very much) to statements such as, “In the last 14 days have you been bothered by feeling fearful?”, with the cut-off of 2 recommended as a valid predictor of mental distress. The SCL-5 has been shown to correlate well with the SCL-25 (r = 0.92). It is designed to screen for global psychiatric morbidity, namely anxiety and depression. The SCL-5 has good internal consistency (Cronbach’s alpha = .80).
- Mental Health Inventory (MHI) [39] consisting of 34 items designed to measure psychological well-being and distress on a 5-point Likert scale that ranges from 1 (all the time) to 5 (none of the time) to statements such as, “Did you feel depressed?”, quantified people’s mental health state during adversity. A higher score indicates better mental health. The MHI has a high level of internal consistency (Cronbach’s alpha = .93).
- Revised Life-Orientation Test – Revised (LOT-R) [40],is a 10 item life orientation test to assess people’s outlook on life measured on a 5-point Likert scale of 0 (strongly disagree) to 4 (strongly agree) to items such as, “I enjoy my friends a lot.”, with higher scores indicating a more pessimistic attitude. The correlation between the original and revised scale is .95. The LOT-R has an acceptable level of internal consistency (Cronbach’s alpha = .72)
- Physical activity was measured using the International Physical Activity Questionnaire short form (IPAQ-SF) [41–43], assessing frequency, intensity and duration of physical activity in days and minutes; a higher self-reported score indicates a higher level of physical activity. The IPAQ has an acceptable level of internal consistency (Cronbach’s alpha = .73) and has been deemed suitable for national population-based prevalence studies of participation in physical activity.
- The Insomnia Severity Index (ISI) [44]. A 7-item measure in which participants respond to statements such as, ‘How satisfied/dissatisfied are you with your current sleep pattern?’. There are a variety of different scales of response, each raw score for the seven items is added to form a total score of sleep quality, the higher the total score, the higher the level of insomnia or lower the sleep quality. The ISI has an appropriate level of internal consistency (Cronbach’s alpha = .84) and a high test-retest reliability (0.84–1) and a strong positive correlation with the Pittsburgh sleep quality index.
- QOL was measured using the WHOQOL-BREF [45], a 26- question short version of the original WHOQOL-100 designed to assess QOL. The response options for each item are rated on a 5-point Likert scale from 1 to 5 to statements such as, “How satisfied are you with yourself?”. The questionnaire splits into four domains of QOL: physical health, psychological health, social relationships and environment, with two questions to reflect overall QOL and general health. Higher scores indicate higher QOL. The WHOQOL-BREF has a high level of internal consistency (Cronbach’s alpha = .89)

### Procedure

The survey was distributed using the link generated by Qualtrics via the social media channels ‘Facebook’ and ‘WhatsApp’, and email. Participants voluntarily opted into the study. Data were coded and analysed using the Statistic Package for the Social Sciences version 25 (SPSS).

### Data analysis

The data was inputted into SPSS from Qualtrics and cleansed, removing any participants who did not complete the survey and checking the survey was transferred appropriately without mistakes. The remaining data were coded and scored and a total score for each participant generated. The normality of distribution and variance were checked via SPSS and we used Pearson’s correlation coefficient, to check the data met the assumptions of each test. Descriptive statistics were produced describing age, exercise categorial level, resilience, QOL, mental health, sleep, and life-orientation. Based upon their responses to the IPAQ during lockdown, participants who increased, decreased, or kept their exercise level the same were classified as progressors, regressors and maintainers respectfully.

A MANOVA was used to compare resilience and QOL life scores, before lockdown and used to compare resilience and QOL during lockdown at three different levels of exercise: low, moderate, and high. Independent sample t-tests compared differences in QOL and resilience scores between people changing exercise levels on each dependant variable. The independent t-tests allowed the authors to report the exercise level change that increased QOL and resilience. ANCOVA was used to test whether mental health and sleep quality moderated the level of exercise on resilience. A t-test compared differences in location on self-reported exercise levels and resilience. Finally, MANCOVA compared the relationship between exercise level and resilience and QOL whilst controlling for sleep quality and mental health.

## Results

### Variable descriptive statistics

Descriptive statistics of participants’ responses are shown Table 1:

**Table 1:** Mean, standard deviation of participants’ scores on each variable

	Age	Exercise before lockdown	Exercise during lockdown	Exercise level change			Resilience	Quality of life		Mental health			Sleep		Life-orientation
				Reducers	Maintainers	Progressors		Before lockdown	During lockdown	Before lockdown	During lockdown	Difference	Before lockdown	During lockdown	
**Mean (SD)**	47.04 (± 18.98)	2.45 (± .65)	2.52 (± .65)	N/A	N/A	N/A	70.2 (± 17.29)	353.27 (± 46.83)	339.15 (± 59.4)	91.37 (± 18.08)	86.81 (± 20.95)	4.56 (± 16.1)	5.91 (± 4.81)	7.45 (± 6.72)	25.34 (±6.26)
**Participant Number**	N/A	N/A	N/A	10	60	15	N/A	N/A	N/A	N/A	N/A	N/A	N/A	N/A	N/A
**Participant Percentage**	N/A	N/A	N/A	11.8	70.6	17.6	N/A	N/A	N/A	N/A	N/A	N/A	N/A	N/A	N/A

A Pearson’s correlation coefficient to measure the relationship strength between resilience and QOL was carried out to check the data met the assumptions of each test. A Pearson’s correlation coefficient between resilience and QOL was statistically significant *p* < .001. Based on a critical skewness value of 1.96 [46], data was normally distributed on all measures except sleep quality (see Table 2).

**Table 2:** Skewness and Kurtosis scores for participants’ responses to each measure

	Resilience	Quality of life		Mental health		Sleep		Life-orientation	Exercise	
		Before lockdown	During lockdown	Before lockdown	During lockdown	Before lockdown	During lockdown		Before lockdown	During lockdown
**Standard error of Kurtosis**	.517	.517	.517	.517	.517	.517	.517	.517	.517	.517
**Kurtosis Statistic**	1.833	.238	−.244	−.227	−.068	−.004	.995	.323	−.445	−.068
**Standard error of skewness**	.261	.261	.261	.261	.261	.261	.261	.261	.261	.261
**Skewness Statistic**	−1.028	−.679	−.674	−.685	−2.49	0.726	0.261	−.890	−.75	−1.01
**Skewness Score**	−3.94	−2.6	−2.58	− 2.62	−2.49	2.78	4.36	−3.41	−2.87	−4.68

MANOVA showed a statistically significant difference between the groups on the combined dependant variables before lockdown, F (2,82) = 4.22, *p* = .003: Wilks’ lambda = .82, partial n^2^ = 0.09. Analysis of each dependant variable, using a Bonferroni adjusted alpha level of 0.17 showed a statistically significant contribution of resilience F (2,82) = 6.65, *p* = .002, partial n^2^ = 0.14 and QOL F (2,82) = 6.62, *p* = .002, partial n^2^ = 0.14. As exercise level (low, moderate, vigorous) before lockdown increased, so did QOL and resilience.

There was a statistically significant difference between the groups on the combined dependant variables before lockdown, F (2,82) = 7.31, *p* < .001: Wilks’ lambda = .72, partial n^2^ = 0.15. Analysis of each dependant variable, using a Bonferroni adjusted alpha level of 0.17 showed a statistically significant contribution of resilience F (2,82) = 11.46, *p* < .001, partial n^2^ = 0.22 and QOL F (2,82) = 8.88, *p* < .001, partial n^2^ = 0.18. As exercise level during lockdown increased, so did QOL and resilience.

A higher QOL was reported by people who progressed an exercise category from low to moderate or moderate to vigorous (mean = 355.47, SD = 52.61) (mean = 335.66, SD = 60.56). A higher resilience score was reported by people who progressed an exercise category (mean = 72.60, SD = 14.45) (mean = 69.69, SD = 17.89). There was no statistically significant difference in QOL (*t* (83) = 1.17, *p* = .244) and resilience scores (*t* (83) = .59, *p* = .56) between progressors and maintainers.

ANCOVA showed there was a statistically significant effect of exercise level *before* lockdown on resilience, F (1, 85) = 4.59, *p =* .013 even when controlling for sleep quality and mental health scores. *During* lockdown, ANCOVA showed there was a statistically significant effect of exercise level on resilience, F (1, 85) = 6.53, *p =* .002 even when controlling for sleep quality and mental health scores.

There were no statistically significant differences in exercise levels (*t* (83) = 1.81, *p* = .07) and resilience scores (*t* (83) = 1.28, *p* = .21) between those in an urban location and those living in a rural location before lockdown.

There was no statistically significant difference in exercise levels and resilience and life orientation *before l*ockdown, F (2,82) = 0.86, *p* = .43: Wilks’ lambda = .98, partial n^2^ = 0.02. Analysis of each dependant variable, using a Bonferroni adjusted alpha level of 0.17 showed no statistically significant contribution of resilience F (2,82) = 1.63, *p =* .206, partial n^2^ = 0.09 and life-orientation F (2,82) = 1.01, *p =* .318, partial n^2^ = 0.01. A one way between-subjects MANCOVA showed there was a statistically significant difference between the exercise levels and resilience and QOL scores even when controlling for sleep quality and mental health scores *before* lockdown, F (2,82) = 2.89, *p* = .024: Wilks’ lambda = .87, partial n^2^ = 0.07. Analysis of each dependant variable, using a Bonferroni adjusted alpha level of 0.17 showed a statistically significant contribution of resilience to exercise levels F (2,82) = 4.59, *p* = .013, partial n^2^ = 0.10, and QOL F (2,82) = 3.79, *p* = .027, partial n^2^ = 0.09. As exercise level before lockdown increased, so did QOL and resilience independent of sleep quality and mental health.

*During* lockdown, a between-subjects one-way MANCOVA showed there was a statistically significant difference between exercise levels and resilience even when controlling for sleep quality and mental health scores, F (2,82) = 3.42, *p* = .010: Wilks’ lambda = .85, partial n^2^ = 0.08. Analysis of each dependant variable, using a Bonferroni adjusted alpha level of 0.17 showed a statistically significant contribution of resilience F (2,82) = 6.53, *p* = .002, partial n^2^ = 0.14, but not QOL F (2,82) = 1.83, *p* = .168, partial n^2^ = 0.04. As exercise level before lockdown increased, so did resilience independent of sleep quality and mental health, whereas QOL was affected by sleep quality and mental health. When the MANCOVA was repeated using the results during lockdown, with just the co-variate of sleep, analysis of each dependant variable, using a Bonferroni adjusted alpha level of 0.17 showed a statistically significant contribution of resilience to exercise level F (2,82) = 10.11, *p* < .001, partial n^2^ = 0.20, and QOL F (2,82) = 4.92, *p* = .010, partial n^2^ = 0.12. As exercise level before lockdown increased, resilience and QOL increased independent of sleep quality but not mental health.

## Discussion

The study investigated how the COVID-19 pandemic affected the mediators and moderators of resilience with respect to exercising. The current study found that as exercise level increases so does resilience. The relationship between exercise and resilience is independent of sleep and mental health under normal conditions. During a pandemic, this relationship is independent of sleep quality, but not mental health. Location does not play a statistically significant role in resilience.

### Resilience and its mediators and moderators

As no exercise intensity was imposed on participants, all exercise was likely to be at the participants’ preferred intensity. This strengthens earlier findings that to increase resilience and QOL the exercise preferred intensity exercise is sufficient [24–26, 47]. A previous study by Carter et al., [26] reported an increase in some QOL domains [26]. The limitation suggested by using a clinical sample, exercise level being lower than the normal distribution [48], was counterbalanced in this research by hypothesis 3 and 6 which controlled for mental health. We found that the relationship between resilience and perceived QOL is independent of mental health under normal conditions but not during a pandemic. The strong *p* value in this study suggests exercising at a higher level has a stronger effect [22, 49–51], perhaps explaining the difference in QOL findings between Carter et al. [26] and this study. Higher intensity exercise being associated with higher self-efficacy [24, 50] could add further insight into explaining this difference, as confidence in body image leads to increased optimism [52, 53], which is correlated with increased resilience. Carter and colleagues’ [26] short intervention time may limit the effects seen [23]. Sustained exercise at a high level being required to exert positive effects is further suggested by our (non-significant findings), replicating the result Dilornzo et al., found, although the non-significant suggests caution in drawing this conclusion. However, this result is more likely to do with a lower than expected and an underpowered study. Our study takes the knowledge of the relationship between exercise, resilience and QOL a step further, suggesting that exercising continuously at a higher preferred intensity increases perceived QOL and resilience, but further research is needed to test this hypothesis.

Contrary to previous literature [54], we found that location does not influence resilience and QOL. The research on how location effects exercise and resilience is in its infancy, with studies having focused on the attachment between the self and environment, drawing on Bowlby and Ainsworth attachment theory [55]. Therefore, research has mostly focussed on infants, and it is unknown whether this can be applied to adult’s attachment with the environment. In-line with our hypothesis on the relationship between location exercise, QoL and resilience, and previous literature [54, 56, 57], this was reversed during lockdown, where more people began to exercise in rural locations and demonstrated higher resilience levels. However, this was not statistically significant. Therefore, the effect of location on exercise and resilience remains unproven. The current study’s non-significant findings in conjunction with the unexpected findings that exercise levels were higher in urban populations before lockdown, demonstrates the need for further investigations into exercise and environment.

### The effect of a pandemic on resilience

Descriptive statistics showed that 17.6% of participants increased their exercise levels during lockdown. The analysis of the findings reported in this study suggests that exercise seemed to become a coping mechanism to moderate resilience. This is demonstrated by resilience increasing with exercise: the pandemic only affecting QOL during lockdown as mental health became a mediator. Exercise becoming a moderator of resilience during a pandemic is further supported by the F value almost doubling in hypothesis 1 during lockdown, showing the results of the pandemic are more than one would expect to see by chance. To the authors’ best knowledge, this provides the first piece of evidence into the effects of exercise on resilience in a pandemic. This is further strengthened by the decreased *p* value demonstrating that the relationship between exercise, resilience and QOL is even more significant during a pandemic with exercise contributing more strongly to the model. The significant *p* value when just the co-variant of sleep was controlled for, suggests that although the pandemic has had a positive effect on resilience it is dependent on mental health, further suggesting the use of exercise to moderate resilience. This is in line with the current knowledge that exercise is often used as a coping mechanism for stress [58–61], shown across student, aging and clinical populations.

### Strengths and limitations

The study is the first of its kind to investigate how a pandemic affects resilience and its moderators and mediators in an under-represented non-clinical population. Despite an opportunistic sample, and a relatively small sample size, data were acceptably normal. As to limitations, the study required participants to remember past states and conditions so a bias may have been introduced in which positive and negative attitudes would be enhanced [62, 63] due to the episodic encoding and retrieval process attaching emotion to each event stored [64–66]. Exercise levels could not be verified, therefore, we relied on people to correctly self-report. The relatively low sample size, not surprising considering the pandemic, does not rule out a type two error for non-significant results. The authors would like to clarify, the limitation of time hindered the opportunity to leave data collection open for longer.

Although exercise as a coping mechanism increases resilience and reduces stress, it has been linked to personality type [61], with those who are extroverted and less neurotic being more likely to exercise. In accordance with social determination theory, extroverts demonstrate more internal motivation [27, 67], being more likely to exercise. The personality type of each participant in this study was not measured, however, distribution of the survey on social media, coupled with extroverts’ increased use of these platforms [68] suggests extroverts are more likely to have completed it.

The authors recommend further studies capturing larger samples, a measure of actual exercise levels, and more longitudinal studies capturing the longer-term impact of the pandemic on the relationships reported in the current study. Future perspectives to support resilience strategies can be carried out once rules on social distancing are relaxed enough to take a measure of actual exercise levels.

## Conclusion

Exercise is strongly correlated to resilience and during a pandemic such as COVID-19 it becomes a mechanism in which to moderate resilience. The relationship between exercise and resilience has been supported by this study. However, the influence that a pandemic had on mental health is mediated by its effect on quality of life.

## Data Availability

All date generated or analysed during this study are included in this published article.
